# Epigenetics: Roles and therapeutic implications of non-coding RNA modifications in human cancers

**DOI:** 10.1016/j.omtn.2021.04.021

**Published:** 2021-05-01

**Authors:** Dawei Rong, Guangshun Sun, Fan Wu, Ye Cheng, Guoqiang Sun, Wei Jiang, Xiao Li, Yi Zhong, Liangliang Wu, Chuanyong Zhang, Weiwei Tang, Xuehao Wang

**Affiliations:** 1 Hepatobiliary/Liver Transplantation Center, The First Affiliated Hospital of Nanjing Medical University, Key Laboratory of Living Donor Transplantation, Chinese Academy of Medical Sciences, Nanjing, Jiangsu, China; 2 Department of General Surgery, Nanjing First Hospital, Nanjing Medical University, Nanjing, Jiangsu, China

**Keywords:** epigenetics, RNA modifications, cancer, non-coding RNAs, immune, target therapy

## Abstract

As next-generation sequencing (NGS) is leaping forward, more than 160 covalent RNA modification processes have been reported, and they are widely present in every organism and overall RNA type. Many modification processes of RNA introduce a new layer to the gene regulation process, resulting in novel RNA epigenetics. The commonest RNA modification includes pseudouridine (Ψ), *N*^7^-methylguanosine (m7G), 5-hydroxymethylcytosine (hm5C), 5-methylcytosine (m5C), *N*^1^-methyladenosine (m1A), *N*^6^-methyladenosine (m6A), and others. In this study, we focus on non-coding RNAs (ncRNAs) to summarize the epigenetic consequences of RNA modifications, and the pathogenesis of cancer, as diagnostic markers and therapeutic targets for cancer, as well as the mechanisms affecting the immune environment of cancer. In addition, we summarize the current status of epigenetic drugs for tumor therapy based on ncRNA modifications and the progress of bioinformatics methods in elucidating RNA modifications in recent years.

## Graphical abstract

**Figure:**
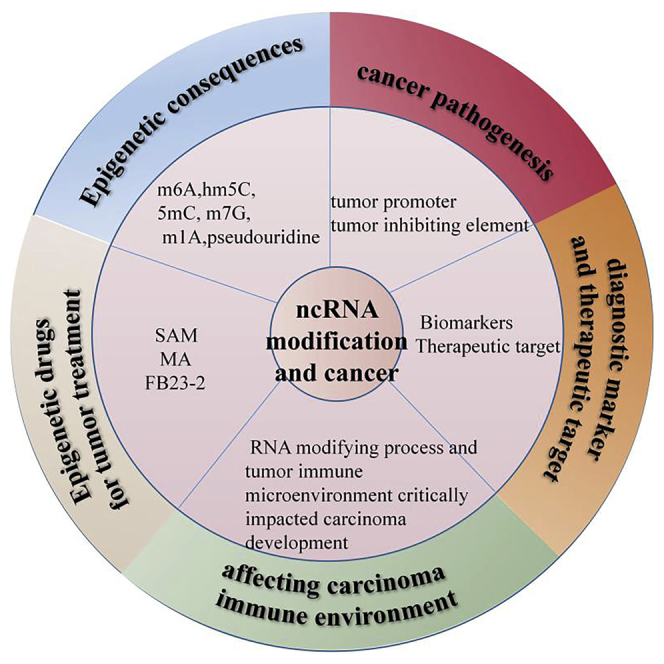

## Teaser

In this review, Rong et al. focus on ncRNAs to summarize the epigenetic consequences of RNA modification, and the pathogenesis of cancer, as diagnostic markers and therapeutic targets for cancer, as well as the mechanisms affecting the immune environment of cancer.

## Introduction

In the last 10 years, changes in gene-expressing state regulation, not involving cell DNA sequence alteration, have aroused major concerns. The mentioned changes indicate “epigenetically related” changing processes, which extensively cover changing processes inside the methylating process of DNA cytosines, changing processes inside chromatin and histone configurations, as well as changing processes inside the expressing state of non-coding RNAs (ncRNAs), controlling the stable characteristic of numerous messenger RNAs (mRNAs), and becoming “master regulating elements” for the gene-expressing state.^1^^,^^2^ Epigenetics provides a molecular explanation that can bridge the gap between genomic and environmental signals during development, and can be linked to lifestyle and environmental conditions during development in the uterus or postpartum. The flexibility of the epigenome represents an attractive opportunity to understand disease variants, and some initiatives to obtain the epigenome of human diseases have been rapidly developed, especially in cancers.^3^^,^^4^

Overall life modes require systems regulating RNA conditions as well as activity. One vital proportion of the mentioned RNA control bands appears under the transcribing process condition, whereas an important proportion pertaining to RNA homeostasis is increasingly dependent on the RNA stable characteristic and degrading process. It is especially true in terms of normal diseases facing humans, e.g., carcinoma, in which abnormal RNA transcriptomes constitute markers of cells under transformation that fail to comprehensively result from changes in the transcription initiation sites related to DNA.^5^^,^^6^ Therefore, the process to map the RNA epigenetic-modifying process is progressively vital for understanding RNA’s various biology-related functions. No less than 160 kinds of chemistry-related modifying processes have been discovered in RNA.^7^ Cellular RNA has a variety of various chemistry-related modifying processes. These modifying processes are engageed in overall RNA metabolism fields.^8^^,^^9^ The commonest RNA-modifying process covers pseudouridine (Ψ), *N*^7^-methylguanosine (m7G), 5-hydroxymethylcytosine (hm5C), *N*^1^-methyladenosine (m1A), 5-methylcytosine (m5C), *N*^6^-methyladenosine (m6A), and others.^10^ There are a number of recent and excellent reviews available on epitranscriptomic RNA modification;^11^^,^^12^ as an example of such modification, Jin et al.^13^ described the effect and system pertaining to *N*^4^-acetylcytidine (ac4C) inside the gene-expressing state and regulating process and proved ac4C’s correlation with different diseases in humans, in particular carcinoma. Investigations of RNA-modifying process functions and the relevant molecular systems attention, boosting tool development for detecting particular modifying processes in transcriptomes and genomes. Developing novel technologies promotes knowledge regarding RNA-modifying processes.^14^^,^^15^ For instance, Chen et al.^16^ established RMDisease, one database regarding genetically related variants capable of affecting RNA modification, which identified a total of 202,307 human single-nucleotide polymorphism (SNPs) probably affecting sites pertaining to eight RNA-modifying categories.

Current studies have pointed out that there are two different ways of RNA modifications: one is to change the structure of modified RNA, and then regulate the interaction between RNA and proteins, blocking or inducing; the other is that modified RNA-binding proteins (RBPs) directly recognize and induce follow-up reactions. Dynamic and reversible methylation on RNA is mediated by RNA-modifying proteins called “writers” (methyltransferases) and “erasers” (demethylases). “Readers” (modified RNA-binding proteins) recognize and bind to RNA methylation sites, which influence the splicing, stability, or translation of modified RNAs.^17^ The binding protein mentioned above is a so-called reader of RNA modification, and only m6A readers are known at present. Gene-specific transcription factors and chromatin-modifying enzymes can further regulate the deposition of m6A in newborn mRNA by rejecting or recruiting m6A writer complex. The SMAD2/3 interactome reveals that transforming growth factor β (TGF-β) controls m6A mRNA methylation in pluripotency.^18^, ^19^, ^20^ Two demethylases, obesity-associated protein (FTO) and AlkB homolog 5 (ALKBH5), act as m6A-modified erasers.^21^^,^^22^

Herein, the roles of epigenetics and the therapeutic implications of ncRNAs are reviewed, including transfer RNA (tRNAs), microRNAs (miRNAs), long non-coding RNAs (lncRNAs), circular RNAs (circRNAs), and ribosomal RNAs (rRNAs) in the regulation in human cancers. We mainly summarize the epigenetic consequences of RNA modification, and the pathogenesis of cancer, as diagnostic markers and therapeutic targets for cancer, as well as the mechanisms affecting the immune environment of cancer. We also summarize the current status of epigenetic drugs for tumor therapy based on ncRNA modifications and bioinformatics methods for RNA-modifying processes, as well as questions to be addressed.

## Epigenetic types of ncRNA modifications

### m6A and ncRNAs

**Figure 1:**
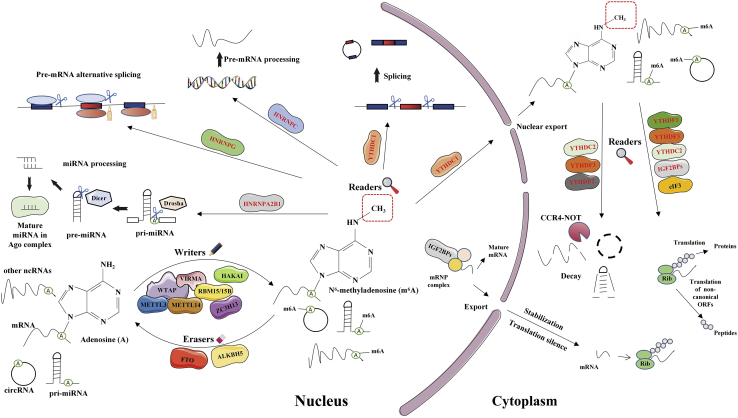
m6A-modifying process on RNAs m6A receives deposition onto RNAs by the m6A writing element, including WTAP, METTL14, METTL3, VIRMA, HAKA1, RBM15/15B, and ZC3H13. m6A can be removed by FTO and ALKBH5. The m6A modification can be identified by readers such as the YT521-B homology (YTH) domain-containing protein family (YTHDC1, YTHDC2, YTHDF1, YTHDF2, YTHDF3), the HNRNP family (HNRNPC, HNRNPG, HNRNPA2B1), and IGF2BPs (IGF2BP1, IGF2BP2, IGF2BP3). In the nucleus, YTHDC1 can regulate RNA splicing and mediate RNA nucleus export; HNRNPC, HNRNPG, and HNRNPA2B1 can mediate pre-mRNA precessing, pre-mRNA alternative splicing, and miRNA processing, respectively. In the cytoplasm, YTHDF1 and eIF3 mediate translation in cytoplasm initiation; YHTDF2 mediates mRNA decay and can mediate cap-independent translation cooperate with eIF3; YTHDC2 mediates RNA translation; and IGF2BPs mediates RNA degradation and stabilization.

m6A modification regulates mRNA at different levels, including structure, maturation, stability, splicing, export, translation, and decay.^23^ The RNA m6A-modifying process presents a methylating process in adenosine N6 site, i.e., the widest internal modification in eukaryotes mRNA.^24^ The RNA m6A-modifying process also undergoes primary catalysis with one complex of methyltransferase (writers) covering methyltransferase-like 14 (METTL14) and methyltransferase-like 3 (METTL3), as well as additional protein subunits, which consist of motif protein 15/15B (RBM15/15B) that binds to RNA, Vir-like m6A methyltransferase with association (VIRMA), zinc finger CCCH-type covering 13 (ZC3H13), and Wilms tumor 1-associating protein (WTAP).^21^^,^^25^ m6A can be demethylated by fat mass and obesity-related protein (FTO) and human AlkB homolog 5 (ALKBH5) (erasers), hence acting as a reversible modification.^22^^,^^26^ The m6A-modifying processes causing RNA structure to vary locally are able to be identified by selection-based RNA-binding proteins (m6A reading elements). The major m6A reading elements cover the family of protein supplemented by the YT521-B homology (YTH) domain (e.g., the cytoplasmic YTHDF3, YTHDF2, YTHDF1, and YTHDC2 and the nuclear *Homo sapiens* YTH domain containing 1 [YTHDC1]).^27^^,^^28^ The rest of the RNA-binding proteins, e.g., nuclear factor κB (NF-κB)-related protein (NKAP) and heterogeneous nuclear ribonucleoprotein (HNRNP) family (HNRNPG, HNRNPC, and HNRNPA2B1), impact the fates of RNAs and the functions exhibited by cells via the particular identification of m6A (Figure 1).^29^ Alarcón et al.^30^ found that HNRNPA2B1, both *in vivo* and *in vitro*, bound to m6A-assuming RNAs; its biochemically related footprint complies with the m6A consensus motif. HNRNPA2B1 directly binds to several nuclear transcripts and can elicit basically the same selective splicing influence as that of the m6A writing element METTL3. In addition, HNRNPA2B1 can promote primary miRNA processing, interact with DGCR8 (the miRNA microprocessor complex protein), and bind to m6A signs inside one subdivided primary miRNA transcript set. As proven by an m6A map analysis, lncRNAs undergo extensive m6A modification as well. The m6A methylating process is an RNA structure-related switching and also engages in the competing endogenous RNA (ceRNA) model mediated by lncRNA.^31^ Numerous m6A locations on circRNAs do not comply with those at mRNAs, regardless of sharing m6A reading elements (YTHDF1/YTHDF2) and writing elements (METTL3/ METTL14) showing interactions with mRNAs. m6A facilitates cytoplasmic export of circRNAs, drives circRNA translation, and mediates circRNA degradation.^32^^,^^33^ Previous reviews have summarized the role of m6A modification in the regulation of ncRNA, including the important processes of controlling RNA functions, including processing, stabilization, and transportation, and the crosstalk between RNA m6A-modified and ncRNA in promoting tumor growth and progression.^34^

### m5C and ncRNAs

**Figure 2:**
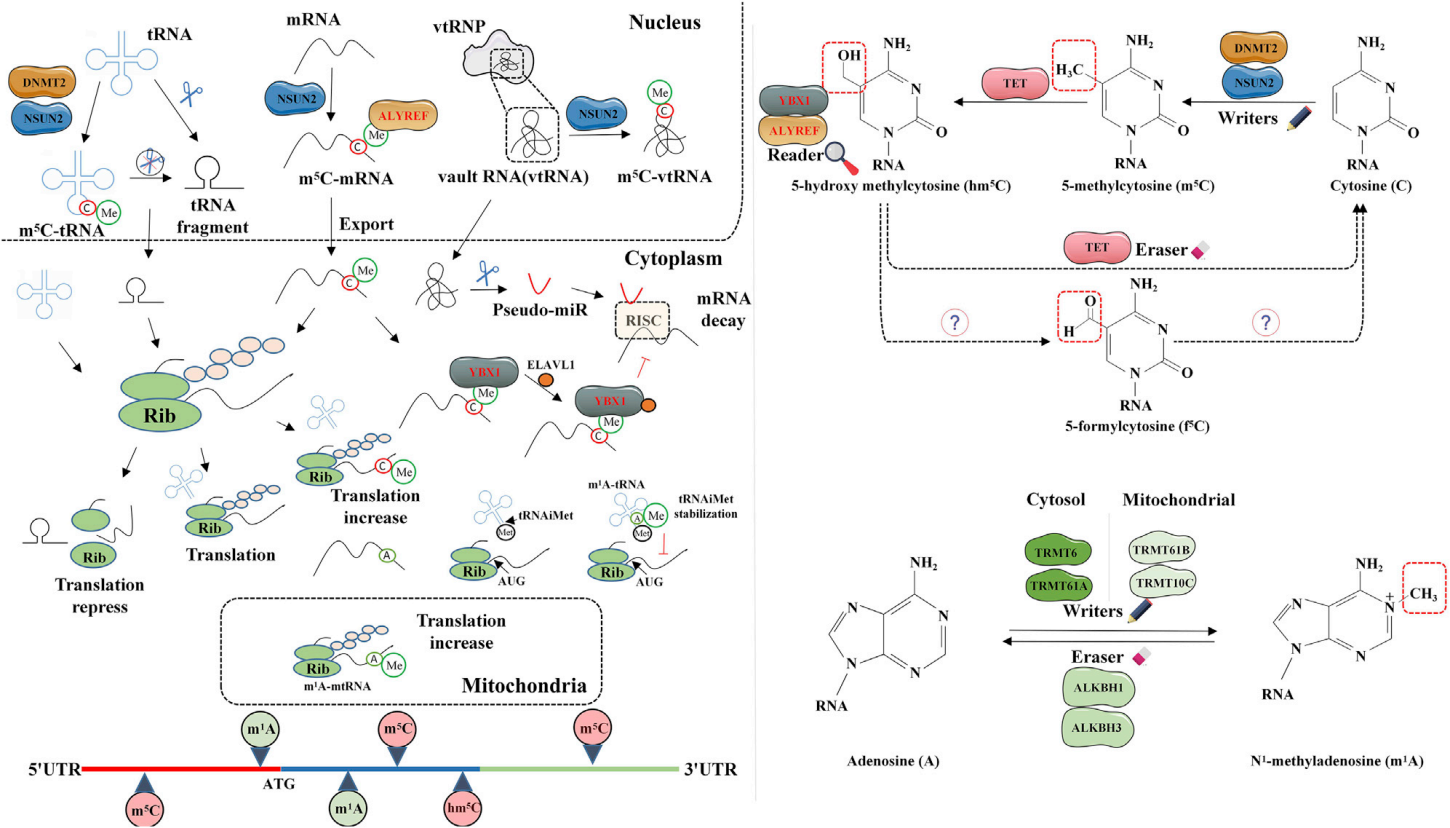
The methylation and demethylation of m5C and m1A in RNAs Through m5C and m1A modification, processes such as translation and mRNA decay can be regulated. ALYREF can recognize m5C and mediate RNA export; DNMT2 and NSUN2 can mediate tRNA m5C modification, preventing tRNA from being degraded into tRNA fragments, thus increasing translation; NSUN2 can mediate vault RNA (vtRNA) m5C modification; and YBX1 can recognize mRNA m5C modification, then combine with ELVAL1. These two processes can inhibit RNA-induced silencing complex (RISC)-mediated mRNA decay. The m1A modification of mtRNA can increase translation, and the m1A modification of tRNA leads to tRNAiMet stabilization, thus suppressing translation.

m5C is also reported to be involved in the regulation of RNA metabolism. m5C is not only detected in ribosomal RNA and metastatic RNA. It was also found that m5C modification in mRNAs was catalyzed by members of the NOP2/Sun RNA methyltransferase family and accumulated significantly near the translation initiation 3′ untranslated region (UTR) site. ALY/REF output factor (ALYREF) was further identified as the m5C-binding protein (reader) mRNA, which in the nucleus promotes the output of m5C-modified mRNAs.^35^, ^36^, ^37^, ^38^, ^39^, ^40^, ^41^ RNA m5C has its formation based on the methylating process under cytosine’s C5 position. It was demonstrated that m5C-modifying processes undergo catalysis with the NOL1/NOP2/SUN (NSUN) domain protein family (NSUN7, NSUN6, NSUN5, NSUN4, NSUN3, NSUN2, and NSUN1) (Figure 2).^29^^,^^42^ Then, Aly/REF export factor (ALYREF), a particular protein-binding mRNA m5C, capable of reading modifying processes, was found to be a m5C reader.^41^ m5C function in rRNA and tRNA is primarily connected with translating control and RNA stability.^43^, ^44^, ^45^, ^46^ Moreover, a m5C structure-related function is evidenced in tRNA.^47^

### m1A and ncRNAs

m1A is found in mitochondrial (mt) transcripts, mRNA, rRNA, and tRNA (Figure 2).^48^, ^49^, ^50^, ^51^ TRMT6 and TRMT61A refer to yeast Trm61 and Trm6 human orthologs, separately, accounting for the m1A58-modifying process of cytoplasmic tRNAs.^52^ As suggested, two ALKB family proteins (i.e., ALKBH3 and ALKBH1) can carry out the demethylation of m1A.^53^^,^^54^ ALKBH3, which is prostate carcinoma antigen-1 (PCA-1), exhibits high expression in several cancers of humans and facilitates angiogenesis and apoptotic resistance in pancreatic and prostate cancer.^55^ It is noteworthy that ALKBH3 was recently reported to be a tRNA demethylase for promoting the protein-synthesizing process in cancer cells.^56^ m1A modification was found on mRNA by transcription and mass spectrometry.^50^^,^^53^ Preliminary estimates based on m1A immunoprecipitation and sequencing show that there are thousands of m1A loci.^50^ The single-nucleotide mapping method gives a more conservative number, ranging from 15 to 500.^51^^,^^57^ The single-nucleotide method identified 215 m1A loci on mRNA27. This method relies on an engineered reverse transcriptase (RT) enzyme to locate m1A.^58^

### m7G and ncRNAs

**Figure 3:**
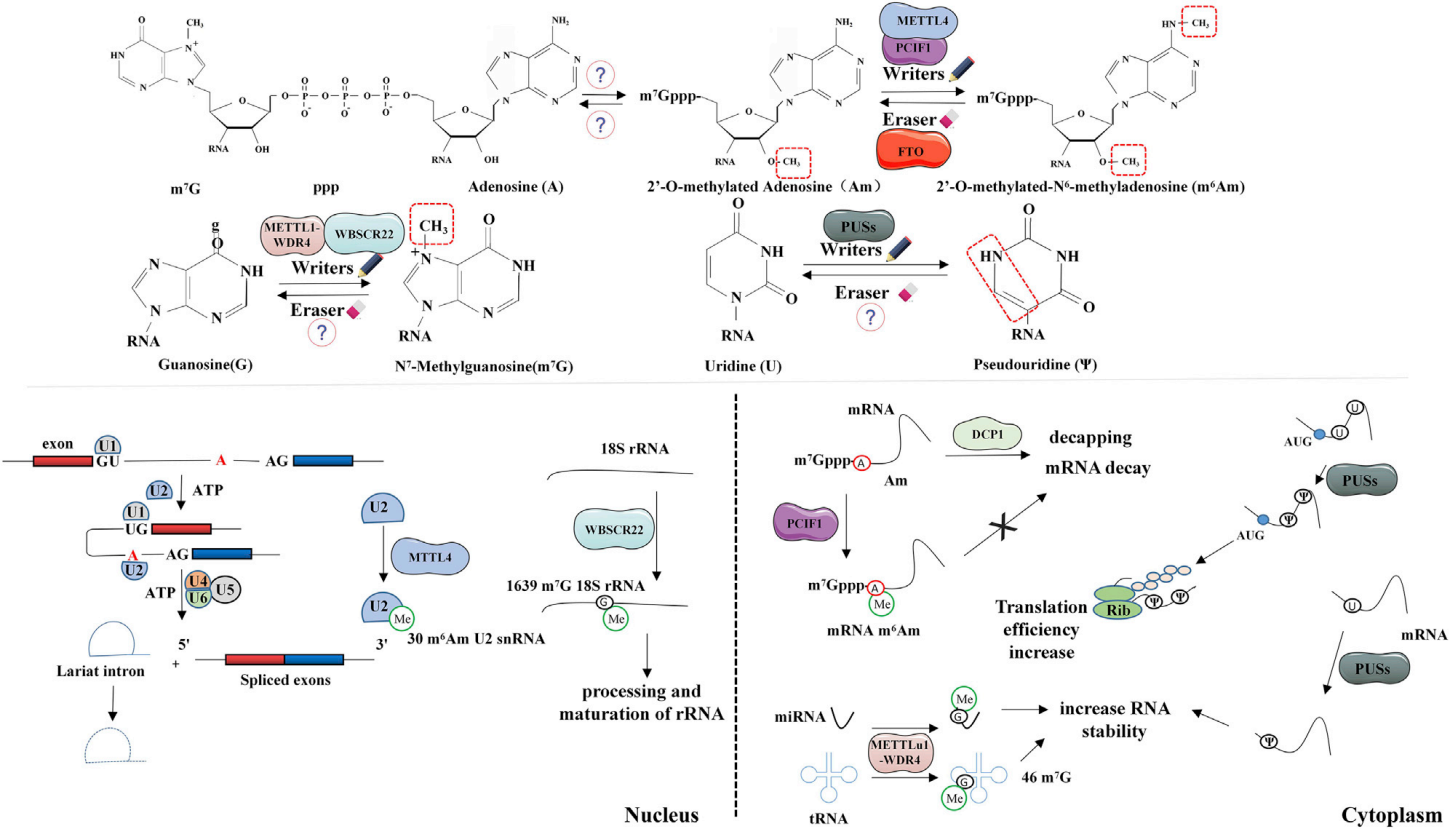
m7G, m6Am, and Ψ modification in RNAs The m7G and Ψ modifications are widespread in cells and cooperate with m6Am modification for engaging in different intracellular biological procedures. In the nucleus, METL4 mediates the m6Am modification of U2 snRNA, which regulates RNA splicing, and WBSCR22 mediates 18S rRNA m7G modification, thereby regulating processing and maturation of rRNA. In the cytoplasm, PCIF1-mediated mRNA m6Am modification can avoid DCP1-mediated modification decapping mRNA decay; METLL1-WDR4 mediated miRNA, tRNA m7G modification and PUSs-mediated mRNA Pesudoganylation can increase RNA stability; in addition, PUSs-mediated mRNA Pesudoganylation can also improve translation efficiency.

m7G exists in the internal sites of tRNA and rRNA. In yeast, small ribosomal subunit (SSU) rRNA, the 1575 site, is m7G-modified by the Bud23-Trm112 heterodimer.^59^^,^^60^ Most bacterial ribosomes are also m7G modified, including *Escherichia coli* ribosomes, which are m7G modified on both SSU and large ribosomal subunit (LSU) rRNA.^61^^,^^62^ Some tRNAs have conservative m7G modification at position 46 to stabilize the tertiary N13-N22-m7G46 folding by improving the geometry of the base triple tRNA.^63^ RNA m7G refers to a notable mRNA cap-modifying process. It exists widely in tRNA. During the past few years, it was also found in mRNA.^64^ METTL1-WDR4, i.e., a tRNA m7G methyltransferase complex, sets up a subset of internal m7G in mRNA.^65^ Nuclear paraspeckle assembling transcript 1 (NEAT1) refers to a lncRNA as one vital subnuclear paraspeckle body structure. In the two isoforms (NEAT1_1 and NEAT1_2) under the generation from the processing of alternative 3′ end RNA, NEAT1_2, which is longer, is critical to the paraspeckle forming process (Figure 3).^29^ NEAT1 RNAs’ 3′ end processing and stability undergo regulation via arsenic resistance protein 2 (ARS2), an element that interacts with the cap-binding complex, binding to RNA polymerase II transcripts’ m7G cap configuration. ARS2 knockdown results in NEAT1_2 being preferentially stabilized. The mentioned results indicate a suppressing effect exerted by ARS2 inside the NEAT1_2-expressing process, as well as the later forming process pertaining to paraspeckles.^66^

### Other RNA-modifying processes and ncRNAs

2′-*O*-methylated adenosine (Am) refers to the first adenosine proximal to the 5′ cap, capable of undergoing in-depth methylation based on methyltransferase PDX1 C-terminal inhibiting factor 1 (PCIF1) for forming m6Am. Consistent with m6A, the *N*^6^-methyl group of m6Am is also capable of undergoing demethylation by FTO (Figure 3).^67^ m6Am can make mRNA stable through the prevention of decapping mRNA 2 (DCP2)-mediated decapping and mRNA decay.^68^ Moreover, ncRNAs refer to m6Am undergoing inner methylation.^69^ Chen et al.^70^ demonstrated that METTL4 is an emerging internal m6Am methyltransferase, mediating the *N*^6^-methylating process of Am30 on U2 small nuclear RNA (snRNA) under an AAG motif *in vitro* and *in vivo*. Their study indicates that the RNA inner m6Am-modifying process undergoes mediation by METTL4; however, whether METTL4 exhibits additional substrates other than U2 snRNA should be studied in depth.

RNA m5C can receive the deeper oxidization from ten-eleven translocating (TET) class enzymes for forming hm5C.^71^^,^^72^ According to Hu et al.,^73^ hm5C shows distribution at lncRNA loci and has a positive correlation to lncRNA transcription. Dysregulated lncRNAs underwent regulation by hm5C in a direct manner or via alternative processes pertaining to characteristic and super-enhancing elements and promoting elements under the modification from hm5C. Moreover, 5hmC participated inside long-range chromatin interactions at lncRNA loci.

ψ, the largest amount of RNA modification process, appears in various RNAs (e.g., rRNA, small nucleolar RNA [snoRNA], snRNA, mRNA and tRNA).^74^^,^^75^ Ψ is critical to precursor (pre-mRNA splicing, snRNP biogenesis, tRNA codon-anticodon base pairing, stress response, translational fidelity and rate, and rRNA folding.^76^ In total, 13 pseudouridine synthases (PUSs, writers) exist, which include dyskerin PUS1 (DKC1), RPUSD4, RPUSD3, RPUSD2, PUS-like 1 (PUSL1), PUS7-like (PUS7L), TruB PSU class member 1 (TRUB1), TRUB2, RNA PSU domain-covering 1 (RPUSD1), PUS10, PUS7, PUS3, and PUS1 (Figure 3).^77^^,^^78^

## Epigenetic consequences of ncRNA modifications

As mentioned above, we have introduced some types of ncRNA modifications, mainly including m6A, m5C, m1A, and m7G, all of which may lead to some epigenetic consequences. For example, METTL3 promotes tumorigenesis of breast cancer (BC), acute myeloid leukemia (AML), and hepatocellular carcinoma (HCC) by enhancing m6A modification.^79^, ^80^, ^81^ METTL14 inhibits the growth and self-renewal of glioblastoma stem cells (GSCs) by enhancing the m6A modification of ADAM19 and reducing its expression in glioblastoma multiforme (GBM).^82^ FTO promotes tumorigenesis by reducing m6A on ASB2 and RARA on the UTR in AML, thus destroying the stability of ASB2 and RARA.^83^ NSUN2 and YBX1 have been shown to drive the pathogenesis of human bladder urothelial carcinoma (UCB) by targeting m5C methylation sites in the hepatoma-derived growth factor (HDGF) 3′ UTR.^84^ The study of Shi et al.^85^ shows that m1A modification and its related regulatory genes play a key role in regulating the progress of HCC. In addition to the role of ncRNA modifications in tumors in the above examples, it can also lead to some other diseases. Abnormal m6A mRNA methylation is associated with the occurrence and development of Alzheimer’s disease.^86^ In addition, m6A modification is also associated with cardio-cerebrovascular diseases. It has been reported that m6A methylation abnormalities play an important role in myocardial hypertrophy, heart failure, aneurysms, vascular calcification, and pulmonary hypertension.^23^ Changes of m5C methylation in CD4^+^ T cells of systemic lupus erythematosus are associated with disease activity.^87^ One study has pointed out that ncRNA modification plays a role in diabetic nephropathy.^88^ Next, we focus on the role of ncRNA modifications in tumors.

### ncRNA modifications and cancer pathogenesis

The effect of ncRNA modifications on carcinoma has a reflection inside the variations of tumor-associated genes. System-based investigation on the cross-link of post-modification regulation, ncRNA modifications, and substrate genes can help indicate the system pertaining to ncRNA modifications inside carcinoma in a comprehensive manner. The present review elucidates the effects exerted by ncRNA modifications in carcinoma pathogenic mechanisms, as shown in Table1.^89^, ^90^, ^91^, ^92^, ^93^, ^94^, ^95^, ^96^, ^97^, ^98^, ^99^, ^100^, ^101^, ^102^, ^103^, ^104^, ^105^, ^106^, ^107^, ^108^, ^109^, ^110^, ^111^, ^112^, ^113^, ^114^, ^115^, ^116^, ^117^, ^118^, ^119^, ^120^, ^121^, ^122^, ^123^, ^124^

**Table 1:** A summary of non-coding RNA modifications in human cancers

Tumor	Modification type	Gene name	Mechanism	Reference
**circRNAs**				

Gastric cancer	m6A	circRNA	m6A methylation promotes the expression of circRNA	^89^
Colorectal cancer	m6A	circ-NSUN2	m6A modification of circ-NSUN2 facilitates cytoplasmic export and stabilizes HMGA2 to promote colorectal liver metastasis	^90^
Cervical cancer	m6A	circ-E7	specific disruption of circE7, which is m6A modified, in CaSki cervical carcinoma cells reduces E7 protein levels and inhibits cancer cell growth both *in vitro* and in tumor xenografts	^91^
Hepatocellular carcinoma	m6A	circ_104075	circ_104075 acts as a ceRNA to upregulate YAP expression by absorbing miR-582-3p; an m6A motif is identified in the 353–357 region of YAP 3′ UTR, and this m6A modification is essential for the interaction between miR-582-3p and YAP 3′ UTR	^92^
Hepatocellular carcinoma	m6A	circ_KIAA1429	circ_KIAA1429 maintains ZEB1 expression through m6A-YTHDF3-ZEB1 and induces cancer cell metastasis	^93^

**lncRNAs**				

Squamous cell carcinoma of the head and neck	m6A	LNCAROD	LNCAROD is stabilized by m6A methylation and promotes cancer progression via forming a ternary complex with HSPA1A and YBX1 in head and neck squamous cell carcinoma	^94^
Ovarian cancer	m6A	lncRNA RHPN1-AS1	m6A reduces RNA degradation and improves the stability of lncRNA RHPN1-AS1, and the RHPN1-AS1-miR-596-LETM1 axis promotes cancer cell proliferation and metastasis	^95^
Ovarian cancer	m6A	MALAT1	the m6A-HNRNPC-mRNA axis promotes cancer cell proliferation	^96^
Hepatocellular carcinoma	m6A	LINC00958	METTL3 mediates m6A upregulation of LINC00958 and upregulation of HDGF expression to promote cancer dipogenesis and progression	^97^
Non-small cell lung cancer	m6A	MALAT1	m6A mRNA methylation initiated by METTL3 directly promotes YAP translation and increases YAP activity by regulating the MALAT1-miR-1914-3p-YAP axis to induce cancer drug resistance and metastasis	^98^
Colorectal cancer	m6A	lncRNA XIST	knockdown of METTL14 increases the m6A level of XIST and reduces XIST expression, enhances the proliferation and invasion of cancer cells, and promotes tumorigenicity and metastasis	^99^
Osteosarcoma	m6A	lncRNA PVT1	ALKBH5-mediated m6A demethylation of lncRNA PVT1 promotes cancer cell proliferation *in vitro* and tumor growth *in vivo*	^100^
Colorectal cancer	m6A	lncRNA GAS5	. lncRNA GAS5 inhibits progression of colorectal cancer by interacting with and triggering YAP phosphorylation and degradation and is negatively regulated by the m6A reader YTHDF3	^101^
Colorectal cancer	m6A	lncRNA RP11	m6A-induced lncRNA RP11 triggers the dissemination of colorectal cancer cells via upregulation of ZEB1	^102^
Pancreatic cancer	m6A	lncRNA KCNK15-AS1	m6A eraser ALKBH5 is downregulated in cancer cells and demethylates lncRNA KCNK15-AS1 and regulates KCNK15-AS1-mediated cell motility	^103^
Colorectal cancer	5hmC	lncRNA	dysreulated colorectal cancer lncRNAs are regulated by 5hmC directly or through abnormal activities of typical and super-enhancers and promoters modified by 5hmC	^73^
Bladder cancer	m5C	lncRNA	the possible link between 5mC modification and differential lncRNAs may relate to enrichment of 5mC reads in the region surrounding super-enhancers of lncRNA	^104^

**miRNAs**				

Glioma	m6A	miR-155/23a	FTO, an m6A RNA demethylase, inhibition enhances the anti-tumor effect of temozolomide by targeting the MYC-miR-155/23a cluster-MXI1 feedback circuit in glioma	^105^
Breast cancer	m6A	miR-146a-5p	METTL14 promotes the migration and invasion of breast cancer cells by modulating m6A and miR-146a-5p expression	^106^
Hepatoblastoma	m6A	miR-186	the miR-186/METTL3/Wnt/β-catenin signaling pathway promotes cancer invasiveness	^107^
Bladder cancer	m6A	miR-221/222	the METTL3/DGCR8-pri-miR-221/222-PTEN axis promotes cell proliferation	^108^
Colorectal cancer	m6A	miR-1246	the METTL3/pri-miR-1246/SPRED2 axis promotes cell migration	^109^
Non-small cell lung cancer	m6A	miR-143-3p	METTL3/miR-143-3p/VASH1-blood-brain barrier promotes the brain metastasis of lung cancer	^110^
Pancreatic cancer	m6A	miR-25	excessive miR-25-3p maturation via m6A stimulated by cigarette smoke promotes pancreatic cancer progression	^111^
Hepatocellular carcinoma	m6A	miR-126	the METTL14/DGCR8-pri-miR-126 axis inhibits cancer metastasis	^112^
Colorectal cancer	m6A	miR-375	METTL14-pri-miR-375 and METTL14-miR-375/YAP1/SP1 inhibit the growth, migration, and invasion of colorectal cancer	^113^
Colorectal cancer	m6A	miR-125b	miR-125b mediates PAR2-induced cancer cell migration by targeting Gab2, and NSun2-dependent RNA methylation contributes to the downregulation of miR-125b by PAR2 signaling	^114^
Breast cancer	m6A	miR-29a-3p, miR-29b-3p, miR-222, miR-1266-5p, miR-1268a, miR-671-3p	a reader of m6A mark, HNRNPA2B1, downregulates expression of miR-29a-3p, miR-29b-3p, and miR-222 and upregulaes of miR-1266-5p, miR-1268a, and miR-671-3p; transient overexpression of HNRNPA2/B1 reduced MCF-7 sensitivity to 4-hydroxytamoxifen and fulvestrant, suggesting a role for HNRNPA2/B1 in endocrine-resistance	^115^
Gastric cancer	m6A	miR-4429	miR-4429 inhibits m6A by targeting METTL3, reduces the stability of SEC62, and prevents the progression of gastric cancer	^116^
Ovarian cancer, hepatocellular carcinoma, and lung cancer	m6A	miRNA	IGF2BP1 promotes the expression of SRF in a conserved and m6A-dependent manner by impairing the miRNA-directed decay of the SRF mRNA in cancer	^117^
Glioblastoma	5mC	miRNA-181a-5p	a significant fraction of miRNAs contains 5mC in glioblastoma cells; cytosine methylation of miRNA-181a-5p is associated with a poor prognosis in glioblastoma patients	^118^
Hepatocellular carcinoma	5hmC	miR-29a	miR-29a induces loss of 5-hydroxymethylcytosine and promotes metastasis of hepatocellular carcinoma through a TET-SOCS1-MMP9 signaling axis	^119^
Prostate cancer	5hmC	miR-29a	global 5hmC modification regulated by miR-29b represses FOXA1 activity; a reduction in 5-hmC activates cancer-related key pathways such as mTOR and androgen receptor	^120^
Colon cancer	m7G	let-7e	METTL1 serves as a tumor suppressor in colon cancer by activating the m7G-regulated let-7e miRNA/HMGA2 axis	^121^
Colon cancer	m7G	miR-149-3p	overexpressed METTL1 increases chemosensitivity of colon cancer cells to cisplatin by regulating miR-149-3p/S100A4/p53 axis	^122^

**tRNAs**				

Tumor	m1A	tRNA	transfer RNA demethylase ALKBH3 promotes cancer progression via induction of tRNA-derived small RNAs	^123^

**rRNAs**				

Hepatocellular carcinoma	m6A	28S rRNA	a new m6A methyltransferase, ZCCHC4, primarily methylates human 28S rRNA and also interacts with a subset of mRNAs; *ZCCHC4* knockout eliminates m6A4220 modification in 28S rRNA, reduces global translation, and inhibits cell proliferation	^124^

### ncRNA modifications function as tumor promoters

ncRNAs plays a key role in the occurrence and development of many cancers. Studies have shown that peptides and proteins encoded by ncRNA, such as HOXB-AS3, FBXW7-185aa, PINT-87aa, and SHPRH-146aa, miPEP-200a, and miPEP-200b, have been proven to play an important role in tumorigenesis and the development of cancer though regulating glucose metabolism, the epithelial-to-mesenchymal transition (EMT), and the ubiquitination pathway.^125^

In HCC, Zuo et al.^97^ reported LINC00958, one lipogenesis-associated lncRNA, with the expressing state increasing inside cell lines and tissues of HCC. Great LINC00958 condition alone assessed weak total surviving state. According to function-related tests, LINC00958 led to the *in vitro* and *in vivo* aggravation of HCC malignant phenotypes. In a mechanistic manner, LINC00958 could sponge miR-3619-5p for increasing the hepatoma-originating growing element (HDGF)-expressing state, helping the HCC lipogenic process and progressing process. The *N*-methyladenosine-modifying process under the mediation of METTL3 generated the LINC00958 upregulating process by the stabilization of its RNA transcript.

Specific to squamous cell cancer of the head and neck (HNSCC), Ban et al.^94^ reported that LNCAROD has a relationship to the advanced T phase and narrowed total surviving state. The m6A-modifying process under the mediation from METTL3 and METTL14 makes LNCAROD stabilized in HNSCC cells. LNCAROD exhibits the primary distribution in the nucleus and binds with Y box binding protein 1 (YBX1) and heat shock 70-kDa protein 1A (HSPA1A) proteins. HSPA1A depletion in LNCAROD-overexpressing cells accelerates proteasomal YBX1 protein degradation.

Specific to bladder carcinoma, according to Han et al.,^108^ METTL3 may have an oncogenic role via the interactive process with DGCR8, the microprocessor protein, and positive modulation for the pri-miR221/222 procedure as dependent on m6A, probably shedding light on bladder cancer therapy.

### ncRNA modifications function to be one tumor-inhibiting element

ncRNA modifications not only plays an important role in promoting tumor progression, but also plays an important role in tumor inhibition. It has been reported that YAP, ALKBH5, IGF2 and other tumor suppressor elements can interact with ncRNA to inhibit tumor progression.

Specific to colon cancer (CC), according to Liu et al.,^121^ et-7e miRNA and METTL1 l exhibited small expressions inside CC cells and tissues in contrast with the relevant common counterparts. METTL1 overexpression suppressed CC cells from proliferating, invading, and migrating, and it facilitated cell death. According to in-depth outcomes, the METTL1 overexpressing process elevated let-7e miRNA inside CC cell lines. In addition, the effects exerted by METTL1 with the overexpressing state onto selected cell functions underwent reversion through let-7e miRNA knockdown. Moreover, HMGA2 acted as let-7e miRNA’s downstream aim, and METTL1 with the overexpressing state suppressed HMGA2-expressing states through let-7e miRNA upregulation inside CC cells. Therefore, METTL1 became one tumor-inhibiting element inside CC through activation of the let-7e miRNA/HMGA2 axis under the regulation of m7G. Interestingly, Liu et al. also found that METTL1-mediated m7G critically impacts the chemoresistance regulating process inside the carcinoma treating process. They built the cisplatin-resistant CC (CR-CC) cells and reported the low expressing state of METTL1 inside CR-CC cells in contrast with the cisplatin-sensitive CC (CS-CC) cells under pairing. In addition, METTL1 with the overexpressing state upregulated the cytotoxic influence on CR-CC cells that was exerted by cisplatin. Furthermore, miR-149-3p acted as the METTL1 downstream target, under the METTL1-positive regulation. Moreover, the influences improving METTL1 with the overexpressing state on CR-CC cell death under the induction of cisplatin received the abrogation through synergistic miR-149-3p knockdown. Also, the S100A4/p53 axis acted as the downstream target of METTL1 and miR-149-3p, and either METTL1 with the overexpressing state or miR-149-3p elevated p53 protein conditions inside CR-CC cells, under the reversion based on the S100A4 increase. On the whole, METTL1 with the overexpressing state could carry out the sensitizing process on CR-CC cells to cisplatin through the miR-149-3p/S100A4/p53 axis modulation.^122^

In colorectal carcinoma (CRC), Ni et al.^101^ found that lncRNA GAS5 has a direct interacting process using YAP’s WW domain for facilitating endogenous YAP’s translocating process (the nucleus to the cytoplasm), as well as facilitating the phosphorylating process and then YAP degradation under mediation from ubiquitin for the *in vitro* and *in vivo* inhibiting CRC progressing process. It is noteworthy that they demonstrated the mA reading element YTHDF3 as an emerging YAP target and one vital YAP signaling player through the facilitation on lncRNA GAS5 degradation under the modification of mA, presenting novel insights for CRC progression.

### ncRNA modifications as diagnostic markers and therapeutic targets

Epigenetics has turned out to be a hotspot in existing scientific research. The RNA-modifying process engages in carcinoma malignancy phenotype regulation through control of the expressing state of carcinoma-associated genes. The uncommon level of the RNA-modifying process facilitates the tumor pathogenic mechanism and progressing process. Although m6A was highlighted extensively, it remains unclear. How RNA modification readers’ expressing state and process receive regulation inside cancer require in-depth studies.

### Biomarkers indicating the occurrence and development of carcinoma

It has been confirmed that ncRNA modifications have become a universal biomarker of carcinoma. By microarray analysis, Zhang et al.^89^ analyzed circRNA expression profiles in adjacent nontumor tissues and poorly differentiated gastric adenocarcinoma (PDGA). Five randomly selected differentially expressed circRNAs (DECs) were verified by quantitative real-time PCR. Using m6A immunoprecipitation and quantitative real-time PCR, m6A qualification of the top 20 DECs was conducted. As revealed from the results, most DECs had m6A-modifying processes, and the trend of m6A-modifying process alteration was mainly consistent with the circRNA expression level. Zhang et al.^92^ identified that circ_104075 was highly expressed in HCC tissues, cell lines, and serum. Mechanistically, HNF4a bound to the −1409 to −1401 region of the circ_104075 promoter to stimulate the expression of circ_104075. Moreover, circ_104075 acted as a ceRNA to upregulate YAP expression by absorbing miR-582-3p. Interestingly, the mA motif was identified in the 353–357 region of the YAP 3′ UTR, and this mA modification was essential for the interaction between miR-582-3p and the YAP 3′ UTR. Furthermore, the diagnostic performance of circ_104075 was evaluated. The area under the curve (AUC) of the receiver operating characteristic (ROC) for circ_104075 was 0.973 with a sensitivity of 96.0% and a specificity of 98.3%. Collectively, they determined that circ_104075 was highly expressed in HCC and elucidated its upstream and downstream regulatory mechanisms. circ_104075 additionally has the potential to serve as a new diagnostic biomarker in HCC. Targeting circ_104075 may provide new strategies in HCC diagnosis and therapy.

### Therapeutic targets of carcinoma

ncRNA modification has been identified to act on carcinogenic driving factors and tumor suppressor factors in a variety of major cancer types. Therefore, a deeper understanding of the complex interaction network coordinated by ncRNA modifications will provide a unique opportunity to design better treatment interventions. Previous studies have reported the presence of m6A, m5C, and pseudo-uracil (ψ) in coding RNA and ncRNA and described their physiological and pathological roles in cancer. These post-transcriptional modifications affect the development, maintenance, and progression of tumors. m6A, m5C, or ψ regulates cell survival in many environments to cope with stress and stem cell function, and targeting them represents a very promising opportunity to specifically target these cell populations and reduce chemotherapy resistance and recurrence.^126^

In CRC cells, the m6A process is suggested to mediate the cytoplasmic export of a crucial oncogenic circNSun2. Chen et al.^90^ indicated that m6A-modified circNSun2 in the nucleus could be recognized by YTHDC1 and exported to the cytoplasm, and circNSun2 then stabilizes high mobility group AT-hook 2 (HMGA2) mRNA by forming the circNSun2/insulin-like growth factor 2 mRNA-binding protein 2 (IGF2BP2)/HMGA2 complex; lastly, CRC cell invasion and liver metastasis are caused. Furthermore, since m6A-modified circNSun2 has been reported to undergo frequent upregulation in tumor tissues and serum samples of patients with CRC with liver metastasis, circNSun2 is likely to be a novel diagnostic/prognostic biomarker as well as a potential therapeutic target for CRCs with liver metastasis.

In HCC, Wang et al.^93^ found that circ_0084922, which came from KIAA1429, named circ_KIAA1429, displayed an increase inside HCC cells and tumor tissues. Overexpression of circ_KIAA1429 can facilitate HCC migration, invasion, and the EMT process. Furthermore, it was demonstrated that Zeb1 was the downstream target of circ_KIAA1429. Upregulation of Zeb1 led to HCC cell metastasis induced by circ_KIAA1429. In addition, YTHDF3 enhanced Zeb1 mRNA stability via an mA-dependent manner. This study revealed that circ_KIAA1429 could accelerate HCC advancement, maintaining the expression of Zeb1 through the mechanism of mA-YTHDF3-Zeb1 in HCC. Next, it was likely to denote one therapeutically related target in HCC.^91^ Additionally, according to Chen et al.,^119^ the miR-29a overexpressing state upregulated the DNA methylating process for the suppressor of cytokine signaling 1 (SOCS1) promoting element and displayed a relationship to *in vitro* and *in vivo* HCC metastasis. Furthermore, miR-29a silenced anti-metastatic SOCS1 through direct TET family targeting, resulting in SOCS1 promoter demethylation inhibition. As proved by the chromatin immunoprecipitation analysis processes, TET1 could change the SOCS1 expressing state via binding to the promoting element area pertaining to SOCS1. Lastly, the miR-29a overexpressing state displayed a relationship to weak clinical outcomes and TET-SOCS1-matrix metalloproteinase (MMP)9 axis silencing inside HCC cases. According to their findings, 5-hmC loss is an epigenetic hallmark of HCC, and miR-29a is an important epigenetic modifier, promoting HCC metastasis through TET-SOCS1-MMP9 axis silencing. The results offer a new strategy for epigenetic carcinoma therapy.

In osteosarcoma (OS), it was found that lncRNA PVT1 expression was upregulated in OS tissues and cells and was significantly related with clinical stage, tumor size, and prognosis of patients with OS. Further investigation revealed that mA demethylase ALKBH5 could associate with PVT1 and suppress its degradation. ALKBH5 decreased the mA modification of PVT1, thus inhibiting the binding of reader protein YTHDF2 in PVT1. Functionally, ALKBH5-mediated PVT1 upregulation promoted the OS cell proliferation *in vitro* and tumor growth *in vivo*. This study suggests that ALKBH5-mediated mA modification of PVT1 contributes to OS tumorigenesis.^100^

## RNA-modifying process affecting the carcinoma immune environment

The RNA-modifying process and tumor immune microenvironment (TME) critically impact carcinoma development. Although immune checkpoint blockade (ICB) therapy has revolutionized carcinoma treatment, many patients do not respond to, or develop, resistance to ICB. m6A in RNA regulates many pathophysiological processes. Li et al.^127^ showed that deletion of the m6A demethylase ALKBH5 sensitized tumors to carcinoma immunotherapy. ALKBH5 has effects on m6A density and splicing events in tumors during ICB. ALKBH5 can modulate the Mct4/Slc16a3 expressing state and the TME’s lactate content, as well as the composing parts of tumor-infiltrating regulatory T cells (Tregs) and myeloid-derived suppressor cells. Notably, a small-molecule ALKBH5 inhibiting element enhanced the efficacy of carcinoma immunotherapy. Notably, the ALKBH5 gene mutation and expression status of melanoma patients correlate with their response to immunotherapy. Li et al.’s results suggest that m6A demethylases in tumor cells increase immunotherapy efficacy, and they found ALKBH5 to be one probable therapeutically related target for enhancing immunotherapy outcome in carcinomas (e.g., CRC and melanoma).

Additionally, Tang et al.^128^ reported that 14 of 21 m6A-related genes were differentially expressed between pancreatic adenocarcinoma (PAAD) and normal tissues in The Cancer Genome Atlas (TCGA)-GTEx cohort. Moreover, an m6A-based model exhibited moderate accuracy in predicting overall survival in PAAD samples. Moreover, they correlated the expression level of m6A-related genes with the immune microenvironment of pancreatic carcinoma for the first time. Specifically, both arm-level gain and deletion decreased the infiltration of CD8^+^ T cells. Overall, these findings suggest a novel anticarcinoma strategy for restoring balanced RNA methylation in tumor cells and guide clinical physicians in developing a new practical approach for considering the impact of related genes on prognosis. Zhang et al.^129^ comprehensively evaluated the mA-modifying process patterns of 1,938 gastric carcinoma samples based on 21 mA regulators, and they systematically correlated these modifying process patterns with TME cell-infiltrating characteristics. The m6Ascore was constructed to quantify mA-modifying process patterns of individual tumors using principal component analysis algorithms. As revealed from the results, three distinct mA-modifying process patterns were determined. The TME cell-infiltrating characteristics under these three patterns display great consistency to the three immune phenotypes of tumors (e.g., immune-desert, immune-inflamed, and immune-excluded phenotypes). They proved that mA-modifying process pattern assessment in single tumors can conduct the prediction of phases regarding tumor inflammatory condition, subdivided types, TME stromal process, genetic change, and case prognostic process. A low m6Ascore, featuring immunity activation and elevated mutation burden, indicated an inflamed TME phenotype, with 69.4% 5-year survival. A high m6Ascore subtype indicated the activation of stroma and lack of an effective immune-infiltrating process, revealing a non-inflamed and immune-exclusion TME phenotype, with poorer survival. A low m6Ascore displayed a relationship with elevated neoantigen load and improved response to anti-PD-1/L1 immunotherapy as well. Two immunotherapy cohorts confirmed that patients with a lower m6Ascore demonstrated significant therapeutic advantages and clinical benefits. As suggested from this study, the mA-modifying process critically affected TME diversity and the complexity forming process. Evaluating the mA-modifying process pattern of individual tumor will contribute to enhancing our cognition of TME infiltration characterization and guiding more effective immunotherapy strategies.

### Epigenetic drugs for tumor treatment based on the ncRNA-modifying process

Given that the RNA-modifying process plays an important role in carcinomas, a chemical that inhibits the RNA-modifying process editing could serve as an effective drug. *S*-adenosylmethionine (SAM) is a universal methyl donor for *S*-adenosylhomocysteine (SAH), receiving the hydrolyzation into homocysteine and adenosine via SAH hydrolase. SAH hydrolase is capable of regulating SAH, and SAH inside cells acts as a competitive inhibiting element for several methyltransferases dependent on adenosylmethionine.^130^ Using a structure-based virtual screening, rhein was found as an inhibiting element of FTO by Chen et al.^131^ Rhein is capable of upregulating m6A’s cellular contents in mRNA through FTO active site binding and avoiding FTO from m6A substrate binding. Moreover, Huang et al.^132^ found that meclofenamic acid (MA) is another highly selective inhibiting element of FTO by competition for FTO binding with m6A-containing substrate. Using structure-based rational design, Huang et al.^133^ created two promising FTO inhibiting element, i.e., FB23 and FB23-2, binding to FTO in a direct manner and suppressing FTO’s mA demethylase process in a selective manner. Mimicking FTO depletion, FB23-2 dramatically suppresses proliferation and promotes the differentiation/apoptosis of human AML cell line cells and primary blast AML cells *in vitro*. Furthermore, FB23-2 noticeably suppresses human AML cell lines from progressing and primary cells inside xeno-transplanted rats. On the whole, as revealed from the information here, FTO refers to one druggable target, and the process to target FTO by small-molecule-inhibiting elements is promising for treating AML.

### Bioinformatics methods for deciphering RNA-modifying process

Information with respect to the positions of RNA-modifying process sites is critical to the characterization of mechanisms and functions of those modifying processes. As impacted by recent advances in genomics and molecular biology, biologists can identify a variety of RNA-modifying processes in an experimental manner. Most currently available high-throughput experimental techniques exhibit limitations due to the following reasons: (1) although certain technology, e.g., methylated RNA immunoprecipitation sequencing (MeRIP-seq), m6A sequencing (m6A-seq), and photo-crosslinking-assisted m6A sequencing (PA-m6A-seq), can be used to identify m6A sites and detect 10s of thousands of m6A-containing sequence fragments in the transcriptome, they cannot locate the exact positions of the m6A sites;^20^^,^^134^^,^^135^ and (2) some techniques, e.g., m6A individual-nucleotide-resolution cross-linking and immunoprecipitation (miCLIP), are capable of detecting m6A sites at the single-nucleotide resolution level, whereas they cannot find a range of RNA-modifying processes with the simultaneous occurrence in the identical RNA molecule.^136^^,^^137^ To address these problems, computational methods can be used in tandem with the experimental identification of RNA-modifying process sites. Many of the mentioned methods have been recently developed to predict RNA modifying process sites. We summarized the prediction tools for m6A and other types of RNA-modifying processes, respectively (Table 2).^138^, ^139^, ^140^, ^141^, ^142^, ^143^, ^144^, ^145^, ^146^, ^147^, ^148^, ^149^, ^150^, ^151^, ^152^, ^153^, ^154^, ^155^, ^156^, ^157^, ^158^, ^159^, ^160^, ^161^, ^162^, ^163^, ^164^, ^165^, ^166^, ^167^, ^168^, ^169^, ^170^ For example, Han et al.^156^ mapped m^6^A peaks in different subcellular components and gene regions. Among those human m^6^A-modifying process, 190,050 and 150,900 peaks were identified in carcinoma and non-carcinoma cells, respectively. Lastly, all results were integrated and imported into a visualized cell-dependent m^6^A database CVm6A. CVm6A specificity is considered to noticeably promote the function and regulation study of the cell-dependent m^6^A-modifying process in disease and developing process. Xuan et al.^157^ designed a comprehensive database, RMBase v2.0 (http://rna.sysu.edu.cn/rmbase/), integrating epitranscriptome sequencing data to investigate post-transcriptional-modifying processes of RNAs, as well as how they are correlated to RNA-binding proteins, disease-related SNPs and miRNA-binding events. From 47 studies among 13 species, RMBase v2.0 expanded with ∼600 datasets and ∼1,397,000 modifying process sites, representing 10-fold expansion nearly in comparison with the existing release. It covers ∼1,373,000 m6A, ∼5,400 m1A, ∼9,600 Ψ-modifying processes, ∼1,000 m5C-modifying processes, ∼5,100 2′-*O*-methylating (2′-O-Me) processes, and ∼2,800 modifying processes of other modifying process types. Moreover, they developed a novel web-based tool termed as “modMetagene” for plotting RNA-modifying process metagenes with the use of a transcript mode. Such a database is conducive to investigating the potential functions and mechanisms of RNA-modifying processes.

**Table 2:** A summary of mA site prediction tools

Modification type	Tool	Link	Reference
m6A	m6Acomet	https://www.xjtlu.edu.cn/biologicalsciences/m6Acomet	^138^
	m6A2Target	http://m6A2target.canceromics.org	^139^
	m6AVar	http://m6Avar.renlab.org	^140^
	iRNA-PseColl	http://lin.uestc.edu.cn/server/iRNA-PseColl	^141^
	WHISTLE	https://whistle-epitranscriptome.com	^142^
	HMpre	https://github.com/Zhixun-Zhao/HMpre	^143^
	iRNA-Methyl	http://lin.uestc.edu.cn/server/iRNA-Methyl	^144^
	pRNAm-PC	http://www.jci-bioinfo.cn/pRNAm-PC	^145^
	RAM-ESVM	http://server.malab.cn/RAM-ESVM/	^146^
	m6Apred	http://lin.uestc.edu.cn/server/m6Apred.php	^147^
	Targetm6A	http://csbio.njust.edu.cn/bioinf/Targetm6A	^148^
	iRNA(m6A)-PseDNC	http://lin-group.cn/server/iRNA(m6A)-PseDNC.php	^149^
	m6APred-EL	http://server.malab.cn/m6APred-EL/	^150^
	DeepFE-PPI	https://github.com/xal2019/DeepFE-PPI	^151^
	m6ATH	http://lin.uestc.edu.cn/server/m6ATH	^152^
	AthMethPre	http://bioinfo.tsinghua.edu.cn/AthMethPre/index.html	^153^
	RFAthm6A	https://github.com/nongdaxiaofeng/RFAthm6A	^154^
	m6AMRFS	http://server.malab.cn/m6AMRFS/	^155^
	CVm6A	http://gb.whu.edu.cn:8080/CVm6A	^156^
	RMBase v2.0	http://rna.sysu.edu.cn/rmbase/	^157^
m5C	iRNAm5C-PseDNC	http://www.jci-bioinfo.cn/iRNAm5C-PseDNC	^158^
	m5C-HPCR	http://cslab.just.edu.cn:8080/m5C-HPCR/	^159^
	RNAm5CPred	http://zhulab.ahu.edu.cn/RNAm5CPred/	^160^
	PEA-m5C	https://github.com/cma2015/PEA-m5C	^161^
	RNAm5Cfinder	http://www.rnanut.net/rnam5Cfinder	^162^
Ψ	PPUS	http://lyh.pkmu.cn/ppus/	^163^
	PIANO	http://180.208.58.66/PSI-WHISTLE/index.html	^164^
	iRNA-PseU	http://lin.uestc.edu.cn/server/iRNA-PseU	^165^
	PseUI	http://zhulab.ahu.edu.cn/PseUI	^166^
	RAMPred	http://lin.uestc.edu.cn/server/RAMPred	^167^
m7G	iRNA-m7G	http://lin-group.cn/server/iRNA-m7G/	^168^
	m7GFinder	https://www.xjtlu.edu.cn/biologicalsciences/m7Ghub	^169^
hm5C	iRNA5hmC	http://server.malab.cn/iRNA5hmC	^170^

## Conclusion and perspectives

As revealed in the collective evidence, RNA modification is progressively critical to the occurrence and development of cancer. Some writers, erasers, and even RNA-modified readers have potential as biomarkers that could serve as drug targets to diagnose and treat cancer. However, existing research on ncRNA and RNA modification is only the tip of the iceberg. Whether ncRNA and RNA modification are normal or specific in cancer needs to be clarified. In particular, we look forward to the relationship between RNA modification and tumor immunity. Therefore, the modification process of ncRNA and its specific targets need to be studied further.

Although many sequencing technologies and databases are essential to predict and study RNA modification, more accurate and faster detection and prediction methods are needed. In addition, we need more information prediction software for ncRNAs and RNA modification sites. After solving the above problems, we expect that scientists can study the correlation between RNA modification and cancer pathogenesis, prognosis, and diagnosis, further explore the mechanism behind its function, design specific small molecules targeting RNA modification, and detect targeted drugs that can penetrate blood to treat cancer by brain barrier.
